# Widespread Terra Firma-Forme Dermatosis: A Rare Presentation

**DOI:** 10.7759/cureus.78875

**Published:** 2025-02-11

**Authors:** Asli Aldemir

**Affiliations:** 1 Department of Dermatology and Venereology, İzmir Katip Çelebi University Faculty of Medicine, Izmir, TUR

**Keywords:** 70% isopropyl alcohol, abnormal keratinization, duncan’s dirty dermatosis, hyperpigmentation, terra firma-forme dermatosis

## Abstract

Terra firma-forme dermatosis (TFFD) is an idiopathic dermatological condition characterized by asymptomatic, dirt-like hyperpigmented brown plaques. The complete disappearance of hyperpigmented lesions upon the application of 70% ethyl alcohol, despite their persistence after routine cleaning with soap and water, serves as both a diagnostic and therapeutic method for the disease. Although only a limited number of case reports have been published in the literature so far, it is a relatively common condition encountered in routine outpatient examinations. Familiarity with this condition is important to prevent unnecessary patient anxiety and to avoid unnecessary laboratory tests and advanced investigations such as skin biopsy. This report presents a case of TFFD in a 44-year-old Turkish male patient who exhibited widespread asymptomatic brownish plaques on the neck, chest, abdomen, back, upper extremities, and thighs. This case is presented both to raise awareness of TFFD and to emphasize its widespread involvement, distinguishing it from previously reported cases.

## Introduction

Terra firma-forme dermatosis (TFFD) was initially identified and described as a unique clinical entity by Duncan et al. [1] in 1987 and is consequently also known as Duncan’s dirty dermatosis. It is characterized by asymptomatic, hyperpigmented patches and plaques with a dirt-like appearance that are resistant to removal through routine washing with water and soap [1]. However, the lesions can be easily eliminated by rubbing with 70% isopropyl or ethyl alcohol gauze pads, which serves as both a diagnostic and therapeutic test for the condition [2]. Although the precise cause of TFFD remains unknown, current theories suggest that impaired keratinocyte maturation leads to the accumulation of melanin in the epidermis [3]. TFFD appears to be a frequently underdiagnosed and underreported dermatological condition, with only a limited number of published cases in the past two decades.

## Case presentation

**Figure 1:**
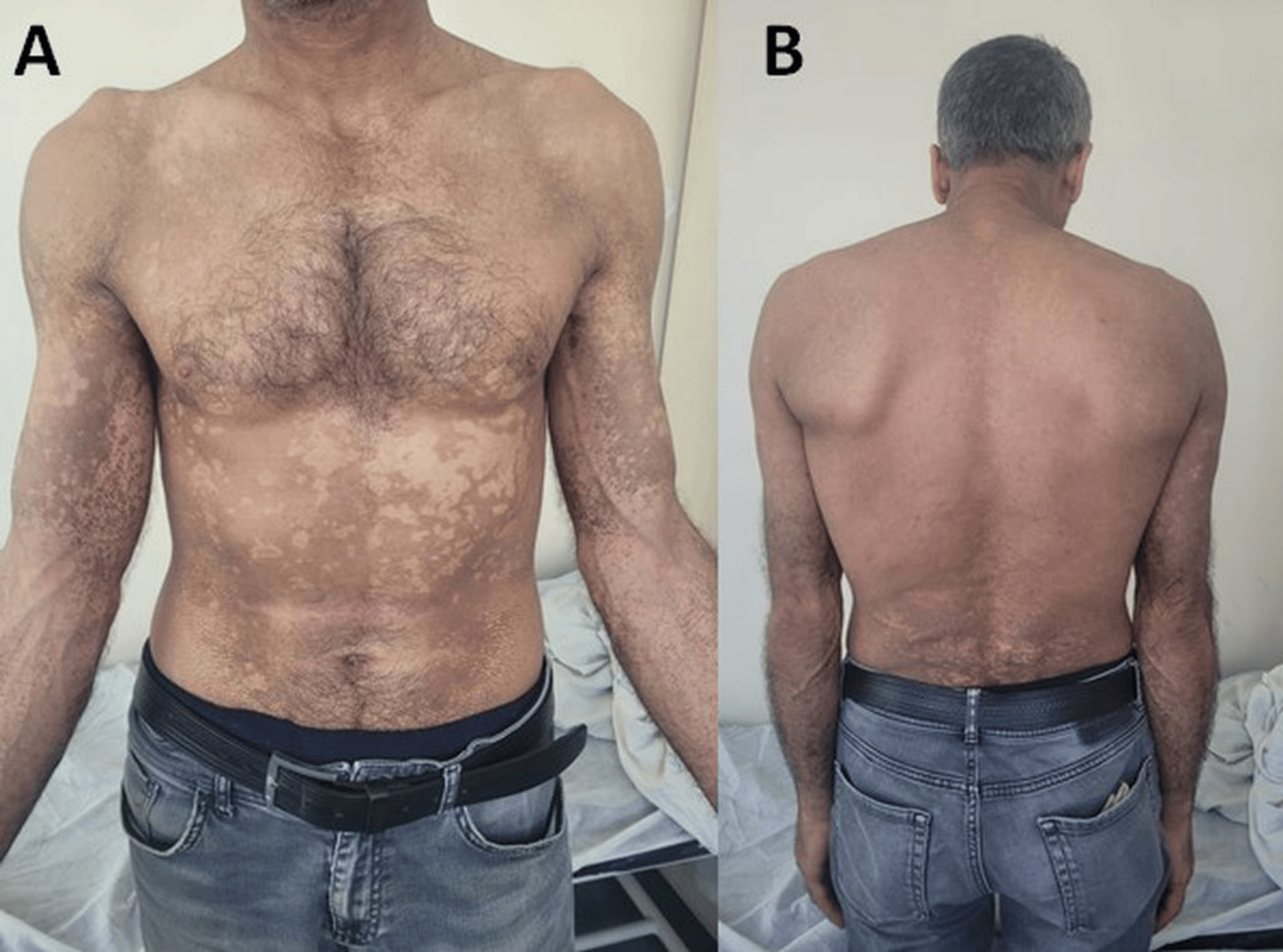
Dermatological examination revealed well-demarcated, irregularly shaped, brownish hyperpigmented plaques with a dirty surface present over the patient's neck, chest, abdomen, bilateral upper extremities (A) and back (B).

A 44-year-old male patient presented to our outpatient clinic with complaints of widespread, asymptomatic brown patches on his body, which he stated had been present for approximately one year. Dermatological examination revealed well-demarcated, irregularly shaped, brownish hyperpigmented plaques with a dirty surface on the neck, chest, abdomen, back, bilateral upper extremities, and thighs (Figure 1). Upon inquiry about personal hygiene, the patient reported taking regular showers and stated that the lesions did not resolve with water and soap. No significant findings were noted in the patient's medical or family history. Laboratory tests requested by a primary care physician the patient had previously visited were within normal limits.

**Figure 2:**
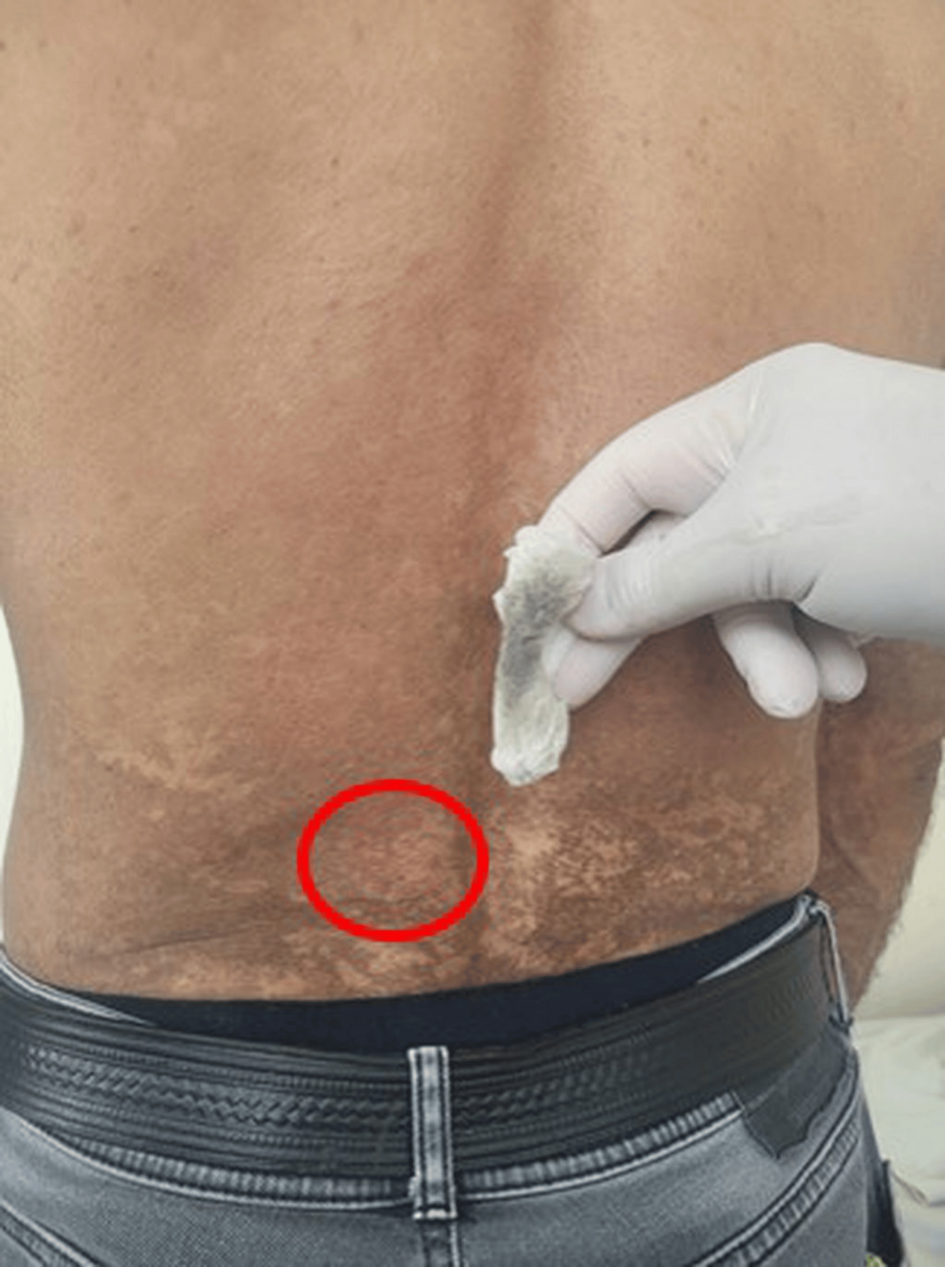
When the patient's lesions were vigorously wiped with 70% isopropyl alcohol-soaked gauze pads, they were immediately and almost completely removed, thereby confirming the diagnosis. The red circle indicates the area wiped with an alcohol-soaked gauze pad, and please pay attention to the color change on the gauze pad.

Due to the widespread distribution of the lesions, a preliminary diagnosis of pityriasis versicolor was considered, and a potassium hydroxide (KOH) examination of skin scrapings was performed, which showed no fungal elements. Additionally, Wood’s lamp examination revealed no yellow-green fluorescence. After that, a preliminary diagnosis of TFFD was considered, and when the patient's lesions were vigorously wiped with 70% isopropyl alcohol-soaked gauze pads, they were immediately and almost completely removed, thereby confirming the diagnosis (Figure 2). When it became clear that all areas could be easily removed with isopropyl alcohol, the patient declined the biopsy. Similar to the literature information, dermatoscopic evaluation of the lesions revealed well-demarcated, brown, polygonal scales arranged in a mosaic-like or cobblestone pattern without significant vascular structures or inflammatory signs [4].

The patient was informed that the lesions should be cleaned using isopropyl alcohol-soaked wipes if they reappear and informed about the importance of regular moisturizing and proper skin care on affected areas for preventing xerosis.

## Discussion

The name “terra firma-forme”, originally derived from Latin, refers to an appearance resembling solid ground, implying that the condition is associated with hyperkeratosis and hyperpigmentation, leading to the characteristic soiled skin appearance [1]. The condition presents as brown to black plaques that can occur at various body sites. The precise etiology of TFFD remains unclear, but the pathomechanism is believed to be related to abnormal keratinization.

In a recent retrospective study by Aslan et al. [5], involving 79 patients, the condition was most prevalent in children (approximately 89% of cases), with an average age of onset of around 10 years and a variability of ±7.5 years. The lesions were commonly located on the trunk and neck, with about 25% of patients also showing involvement of the extremities and fewer than 3% experiencing lesions on the head. While most patients had only one lesion, approximately 30% had multiple lesions.

The differential diagnosis includes dermatosis neglecta which is typically removed with routine bathing [6]. In the differential diagnosis of acanthosis nigricans, the fact that the dark velvety thickening of the skin or "dirt" cannot be removed by normal washing with soap and water, alcohol swabs, or cotton balls, but instead requires keratolytic agents serves as a key distinguishing feature [6,7]. Pityriasis versicolor, caused by Malassezia furfur, was initially considered in the differential diagnosis of our presented case. It exhibits yellow-green fluorescence under a Wood lamp examination, while TFFD remains non-fluorescent [8]. Clinical symptoms are also important in distinguishing between the two; while TFFD is asymptomatic, pityriasis versicolor may be accompanied by mild itching and scaling [8]. Additionally, TFFD can resemble postinflammatory hyperpigmentation; however, a Wood lamp can aid in differentiation by highlighting the enhanced epidermal pigmentation characteristic of TFFD [8]. Additional differential diagnoses to consider are confluent and reticulated papillomatosis, epidermal nevi, melasma, solar lentigo and seborrheic keratosis.

Typically, TFFD is treated and completely resolved immediately after rubbing with a gauze soaked in 70% isopropyl alcohol. This quick clearance with isopropyl alcohol, unlike water and soap, can serve as a diagnostic sign [9]. However, using alcohol with a swab may cause skin irritation [10]. Therefore, combining chemical peeling with an alcohol base could be a preferable approach for achieving optimal cosmetic results [10].

## Conclusions

TFFD is considered a relatively common yet underdiagnosed condition. Raising awareness of TFFD is crucial, as its lesions can be effortlessly removed by rubbing with 70% alcohol, thereby eliminating the need for unnecessary lab tests, skin biopsies, further referrals or investigations. In addition, failure to recognize this disorder may result in unwarranted patient anxiety. Physicians should familiarize themselves with this condition, which appears to be more prevalent than current literature suggests. This case is presented due to the limited number of reported cases in the literature. Additionally, the involvement of almost the entire body, including uncommon areas, in the case presented in this report contributes to the literature, as no similar cases have been previously reported.
